# Applicability of the Greulich–Pyle Method in Assessing the Skeletal Maturity of Children in the Eastern Utter Pradesh (UP) Region: A Pilot Study

**DOI:** 10.7759/cureus.10880

**Published:** 2020-10-10

**Authors:** Praveen K Tiwari, Mayank Gupta, Ashish Verma, Surender Pandey, Amit Nayak

**Affiliations:** 1 Forensic Medicine, Institute of Medical Sciences, Varanasi, IND; 2 Radiology, Institute of Medical Sciences, Varanasi, IND; 3 Anatomy, Institute of Medical Sciences, Varanasi, IND

**Keywords:** greulich- pyle atlas, age estimation, radiological assessment, somatic maturity, population

## Abstract

Background

In forensic proficiency, the distinct model applied for age estimation includes physical examination, i.e., evaluation of somatic maturity and post pubescence peculiarities, dental development, and radiological assessment of skeletal maturity usually of the hand and wrist. The Greulich and Pyle (GP) method of skeletal age (SA) determination is considered quicker and easier with a lower error percentage of prediction. The specificity and applicability of the GP atlas have been recapitulated in many studies. This study aims to assess the applicability and reproducibility of the GP atlas on a sample of the eastern Utter Pradesh (UP) population.

Results

Considering the whole study population, the SA of the male subjects was retarded by 0.89 years or 9.03 months, whereas the SA of females were retarded by 0.36 years or 4.33 months than the chronological age, respectively.

Conclusion

According to this study, it is concluded that the GP atlas may not be applicable for both males and females in the eastern Uttar Pradesh region. The factors responsible for delayed skeletal growth and maturation may vary depending on demographics, ethnicity, and genetics. Further, a detailed study will be conclusive on a greater population size assessing more accurate and precise insights on the applicability and reproducibility of GP atlas.

## Introduction

Estimation of skeletal maturity in a living population plays an important role in establishing diagnosis and treatment protocols for many clinical abnormalities, such as metabolic and endocrine disorders.

In forensic proficiency, the distinct modus applied for age estimation includes a physical examination, i.e., evaluation of somatic maturity and post pubescence peculiarities, dental development, and radiological assessment of skeletal maturity usually of the hand and wrist.

Principally, the two standard methods for skeletal age estimation are:

Bone-by-bone method which incorporates score method by Tanner and Whitehouse, i.e., TW1, TW2, and TW3 and FELS method by Roche et al. [1, 2].

Atlas method comprises the radiological atlas of Greulich and Pyle (GP) [3].

The GP method of skeletal age determination is considered quicker and easier and has a small error of prediction. The specificity and applicability of the GP atlas have been recapitulated in many studies. The atlas is based on a standard hand radiograph directed by the Brush foundation study of human growth and development. This study was performed on Cleveland Caucasian children aged between 0-19 years and most representatives of 100 X-ray images were selected as a standard for each age group [3]. The maturity centers of age estimation can be seen easily in wrist X-ray as it is most rapid, impartial and involves minimal radiation exposure to the subject [4].

To the best of our knowledge, no study has been conducted in eastern Uttar Pradesh referring to the GP atlas. As the age of appearance of maturity centers varies on climatic, topographical, and phylogenetical factors, this study aims to assess the applicability and reproducibility of GP atlas on a sample of eastern Utter Pradesh population.

## Materials and methods

This prospective study was performed between June 2019 and January 2020. Children and young adolescents between 1 and 19 years who were recommended for an X-ray of the wrist were included. A total of 70 wrist radiographs (37 male and 33 female) of random patients were collected. The exact chronological date of birth of subjects and proper consent were obtained to include their radiograph in the study. Subjects with any chronic bone disease, bone injuries, or any metabolic or hormonal disorder were excluded from the study.

**Figure 1:**
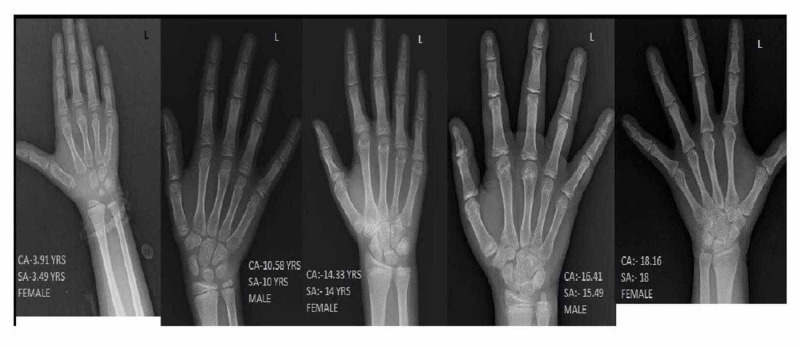
X-ray images of left hand of different chronological and skeletal age in both sex

For bone age determination, a PA view of wrist X-ray was selected, preferably of the left hand and if not available a right-hand radiograph was included in the study as there is a minute, insignificant difference between the right and left hand (Figure 1) [5]. The radiographs were separated into four subgroups according to chronological age (CA) (0-5, 5-10, 10-15, 15-19 years) and sex. The radiographs were interpreted for SA according to the GP atlas by blinded review of three readers (one radiologist, one anatomist, and one forensic medicine expert) competent to assess the methodology of the GP atlas. The radiographs were separated according to sex, and the chronological age was hidden to reduce interobserver error. The positive difference between CA and SA denotes delayed growth whereas the negative difference was considered early growth.

The ethical committee of institute provided the ethical approval for the study with the reference no Dean/2018/EC/930.

Statistical analysis

The mean chronological age was compared to the mean skeletal age, and the difference between CA and SA was calculated for each age subgroup and sex group. The standard deviation of the mean (CA-SA) was calculated. A paired sample t-test was applied, and the p-value was calculated. Statistical analysis was performed using SPSS version 25 (IBM Corp., Armonk, NY).

## Results

**Figure 2:**
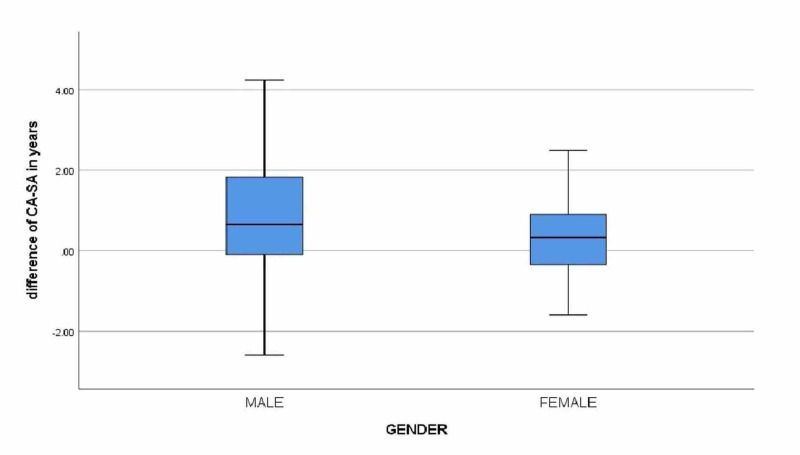
Box plot of difference between chronological age and skeletal age by sex CA: Chronological age; SA: Skeletal age.

In all, 70 subjects’ X-rays were examined, of which 37 (52.85%) were males and 33 (47.15%) were females. The mean chronological age of all subjects was 10.88 ± 4.66 years (SE = 0.55), whereas the mean skeletal age was 10.31 ± 4.82 years (SE = 0.57). The mean difference between CA and SA of all the subjects was 0.56 ± 1.33 years (SE = 0.15, t = 3.5, p = 0.001). Figure 2 represents the radiographs examined using the GP atlas and the difference between the CA and SA of both sexes in different age groups.

In Figure 2, the box plot represents the normal distribution and shows the center and spread of data according to the sex of all 70 subjects studied. The whiskers represent the maximum and minimum values of CA-SA for both males and females.

More specifically, the mean CA of male subjects was 10.33 ± 4.44 years (SE = 0.73), and the mean SA was 9.57 ± 4.90 years (SE = 0.80), and the difference between the CA and SA for males was 0.75 years or 9.03 months with a highly significant p-value of 0.005 (SE = 0.25, t = 2.98 (df = 36), p ≤ 0.05), whereas the mean CA of female subjects was 11.50 ± 4.82 years (SE = 0.83), and the mean SA was 11.14 ± 4.68 (SE = 0.81), and the difference between the CA and SA for females was 0.36 years or 4.33 months with a significant p-value of 0.05 (SE = 0.18, t = 2.00 (df = 32), p ≤ 0.05) as shown in Table 1. Therefore, the SA of the males is retarded by 0.89 years or 9.03 months, whereas the SA of females is retarded by 0.36 years or 4.33 months compared to the chronological age, respectively.

**Table 1:** Gender, chronological age and skeletal age of individual cases

CASE NO.	GENDER	CHRONOLOGICAL AGE	SKELETAL AGE
1	M	1.083	0.74
2	M	1.16	0.74
3	M	3.33	3
4	M	3.24	2
5	M	3.33	1.49
6	M	4.16	2
7	M	4.91	5
8	M	7.24	5
9	M	7.24	7
10	M	7.91	8
11	M	8.16	5
12	M	8.83	7
13	M	9.16	11.49
14	M	10.41	11
15	M	10.49	12.49
16	M	10.58	10
17	M	10.24	6
18	M	11.16	8
19	M	11.66	11
20	M	11.49	8
21	M	11.66	11
22	M	11.83	13
23	M	12.16	11.00
24	M	12.16	11.00
25	M	12.41	11.00
26	M	12.58	13.00
27	M	12.58	12.49
28	M	12.16	11.00
29	M	12.83	11.00
30	M	13.66	13.00
31	M	13.58	13.49
32	M	14.83	15.49
33	M	16.41	15.49
34	M	16.41	19.00
35	M	16.24	15.49
36	M	16.49	14.00
37	M	18.49	19.00
38	F	1.16	1.00
39	F	2.91	4.16
40	F	3.58	4.16
41	F	3.41	2.49
42	F	3.91	3.49
43	F	6.91	6.83
44	F	7.49	7.83
45	F	7.91	6.83
46	F	8.41	8.83
47	F	9.16	8.83
48	F	9.49	7.83
49	F	10.49	10.00
50	F	10.16	11.00
51	F	11.16	11.00
52	F	11.58	12.00
53	F	11.91	12.00
54	F	12.66	14.00
55	F	12.58	12.00
56	F	13.33	13.00
57	F	13.16	11.00
58	F	13.91	13.00
59	F	13.41	12.00
60	F	14.33	14.00
61	F	15.41	17.00
62	F	15.49	16.00
63	F	15.91	13.49
64	F	16.33	16.00
65	F	16.33	14.00
66	F	16.49	14.00
67	F	16.66	16.00
68	F	17.16	17.00
69	F	18.16	18.00
70	F	18.83	19.00

Table 2 describes the mean chronological age, skeletal age, and the p-value of difference means of different age groups according to their sex. As mentioned in Table 3, the males were in the 0-5 year age group (0.89 ± 0.85 years, p = 0.03), and 10-15 year age group (0.81 ± 1.57 years, p = 0.03) had a significant difference between the CA and SA with skeletal retardation of 0.89 years (10.72 months) and 0.81 years (9.75 months), respectively, whereas no significant difference (p ≥ 0.05) was noted in the case of females in any age subgroup.

**Table 2:** Mean chronological age and skeletal age in respective sex CA: Chronological age; SA: Skeletal age.

GROUP	MEAN (CA)	SD	MEAN (SA)	SD	MEAN of (CA-SA)	SD of CA-SA	p-value
Male (n = 37)	10.33	4.44	9.57	4.90	0.753	1.53	.005
Female (n = 33)	11.50	4.82	11.14	4.68	0.364	1.04	.05

**Table 3:** Comparison of chronological age, skeletal age and difference between CA-SA in age subgroup CA: Chronological age; SA: Skeletal age.

Age subgroup	Chronological age (Mean ± SD)		Skeletal age (Mean ± SD)		Difference CA-SA (Mean ± SD)			
	MALE	FEMALE	MALE	FEMALE	MALE			FEMALE
0-5 Years (n = 12)	3.03 ± 1.43	2.99 ± 1.08	2.13 ± 1.48	3.06 ± 1.33	0.89 ± 0.85			-0.06 ± 0.85
					p-value		0.03 ≤ 0.05	0.87 ≥ 0.05
5-10 Years (n = 12)	8.09 ± 0.79	8.22 ± 0.98	7.24 ± 2.39	7.83 ± 0.89	0.84 ± 1.97			0.39 ± 0.82
					p-value	0.34 ≥ 0.05		0.28 ≥ 0.05
10-15 Years (n = 31)	12.02 ± 0.94	12.44 ± 1.38	11.20 ± 2.18	12.08 ± 1.24	0.81 ± 1.57			0.36 ± 1.07
					p-value	0.03 ≤ 0.05		0.26 ≥ 0.05
15-19 Years (n = 15)	16.80 ± 0.94	16.67 ± 1.10	16.59 ± 2.27	16.04 ± 1.08	0.21 ± 1.89			0.62 ± 1.37
					p-value	0.81 ≥ 0.05		0.18 ≥ 0.05

## Discussion

In recent years, much research has been conducted to test the applicability and reliability of the Greulich and Pyle method, as it is the most common and widely used atlas method for bone age determination. As far as we are aware, this is the first study conducted to test the Greulich and Pyle method on the eastern Uttar Pradesh population.

This study reveals that the mean estimated skeletal age of males in the eastern UP is retarded by 0.75 years compared with their chronological age, whereas the mean skeletal age of females is retarded by 0.36 years compared with their chronological age. However, a statistically significant difference was observed in the age subgroups of 0-5 years and 10-15 years, for males in which the skeletal age was retarded by 0.89 years and 0.81 years, respectively, from the chronological age whereas no statistically significant difference was noted in any age subgroup of females due to the very small sample size.

Patil et al. assessed bone age in 375 subjects in the population of Maharashtra between the ages of 1 day and 19 years and compared them to the Greulich and Pyle atlas. It was concluded that the skeletal age retardation was 0.7 years for males and 0.33 years for females from their mean chronological age [6].

Schmidt et al. tested the applicability of the GP method and Thiemann-Nitz method in a German population using 649 hand X-rays of aged 1-18 years and concluded that the mean skeletal age was retarded by -0.49 years for males and -0.39 years for females compared to the chronological age using the GP atlas [7].

Garamendi et al. studied the reliability of methods of forensic age estimation on the Moroccan population of samples for which age estimation was required. The different tests performed were a general physical examination; carpus X-ray for the GP method and dental orthopantomography to determine the degree of maturity of the third inferior molar (Demirjian’s method). They concluded that the carpus X-ray method (skeletal age) was the most useful method than Demirjian’s method (dental age) for age prediction in subjects older or younger than 18 years [8].

According to Loder et al. the GP skeletal age standard for black and white children does not apply to all children today as these standards were derived from white Caucasian children of upper socioeconomic status during 1930. The study was conducted on both black and white children aged between 0-18 years. The author summed up stating that the standards applied to all ages of white girls and black boys up to late childhood and adolescence but not applicable to black girls or any other age groups [5].

Furthermore, Ontell et al. conducted a study to assess bone age in children of diverse ethnicities using GP age standards of 599 white, black, Asian and Hispanic boys and girls and concluded that the GP standards of skeletal age must be used with reservation, particularly in black and Hispanic girls in late childhood and adolescence in Asian and Hispanic boys, when skeletal age may exceed chronological age by 9 months to 11 months 15 days [9].

In one of the Indian studies, Maniar et al. contemplated the effect of malnutrition on hand bones of Indian children and concluded that the upper-income group boys aged between 4 and 8 years are behind by 2 years to the GP standards whereas the girls of the upper-income group closely follow the GP standards except for a lag between 3 and 5 years [10].

This study was deficient in many factors such as physical examination including the height and weight of the subjects, and nutritional and socioeconomic parameters. Finally, further study on a larger population is required including these criteria assessing the applicability and reproducibility of GP atlas on a sample of eastern Utter Pradesh population.

## Conclusions

According to this study, it can be concluded that the Greulich and Pyle atlas may not be applicable for both males and females in the eastern Uttar Pradesh region. Skeletal growth and maturation are more delayed in males than in females. The factors responsible for delayed skeletal growth and maturation may vary depending on demographics, ethnicity, and genetics. Further, a detailed study will be conclusive on a greater population size assessing more accurate and precise insights on the applicability and reproducibility of GP atlas.
